# Cellular and Microbial In Vitro Modelling of Gastrointestinal Cancer

**DOI:** 10.3390/cancers16173113

**Published:** 2024-09-09

**Authors:** Kristina Žukauskaitė, Melissa Li, Angela Horvath, Sonata Jarmalaitė, Vanessa Stadlbauer

**Affiliations:** 1 Department of Gastroenterology and Hepatology, Medical University of Graz, 8036 Graz, Austria; 121279@fhwn.ac.at (M.L.); angela.horvath@medunigraz.at (A.H.); vanessa.stadlbauer@medunigraz.at (V.S.); 2 Institute of Biosciences, Life Sciences Center, Vilnius University, 10257 Vilnius, Lithuania; sonata.jarmalaite@nvi.lt; 3 Biotech Campus Tulln, Fachhochschule Wiener Neustadt, 3430 Tulln, Austria; 4 Center for Biomarker Research in Medicine (CBmed GmbH), 8010 Graz, Austria; 5 National Cancer Institute, 08406 Vilnius, Lithuania

**Keywords:** gut microbiome, in vitro model, disease model, bioreactor system, gastrointestinal tract, intestinal model

## Abstract

**Simple Summary:**

This review aims to improve our understanding of gastrointestinal tract cancer and the side effects of cancer treatment by using advanced in vitro systems. Traditional models like cell cultures and animal studies provide valuable insights but have limitations in replicating the complexity of human disease and raise ethical concerns. By focusing on bioreactor-based in vitro systems, which can mimic the physical and chemical environment of the gastrointestinal tract, this study aims to provide more accurate models for studying cancer and its treatment side effects. These advancements could lead to better insights into disease mechanisms, potentially improving treatment strategies and benefiting the broader research community.

**Abstract:**

Human diseases are multifaceted, starting with alterations at the cellular level, damaging organs and their functions, and disturbing interactions and immune responses. In vitro systems offer clarity and standardisation, which are crucial for effectively modelling disease. These models aim not to replicate every disease aspect but to dissect specific ones with precision. Controlled environments allow researchers to isolate key variables, eliminate confounding factors and elucidate disease mechanisms more clearly. Technological progress has rapidly advanced model systems. Initially, 2D cell culture models explored fundamental cell interactions. The transition to 3D cell cultures and organoids enabled more life-like tissue architecture and enhanced intercellular interactions. Advanced bioreactor-based devices now recreate the physicochemical environments of specific organs, simulating features like perfusion and the gastrointestinal tract’s mucus layer, enhancing physiological relevance. These systems have been simplified and adapted for high-throughput research, marking significant progress. This review focuses on in vitro systems for modelling gastrointestinal tract cancer and the side effects of cancer treatment. While cell cultures and in vivo models are invaluable, our main emphasis is on bioreactor-based in vitro modelling systems that include the gut microbiome.

## 1. Introduction

The human digestive system comprises the mouth, pharynx, oesophagus, stomach and small and large intestines. The gastrointestinal (GI) tract is a tubular structure composed of a lumen, mucosa, submucosa, muscularis and serosa [1,2]. The small intestine comprises three segments, the duodenum, jejunum and ileum, which are primarily involved in breaking down food, absorbing nutrients and water, and propelling food through the gastrointestinal tract. In contrast, the large intestine consists of the cecum, colon, rectum and anal canal. It serves as an organ for absorption, storing waste and transporting solid waste [3].

**Figure 1:**
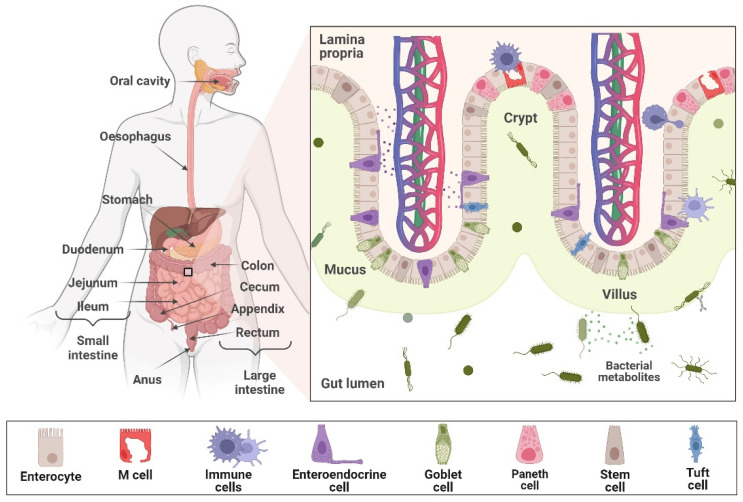
Graphical representation of the structure of the small intestine epithelium. Created with BioRender.com. The illustration is partially based on [7].

The intestinal epithelium is a specialised tissue that ensures the protective barrier function and shields the body from harmful microorganisms and food antigens [4]. It is the most rapidly renewing epithelium in the body, with a complete renewal occurring every 4–5 days [5]. The small intestinal epithelium is structured with finger-like protrusions, called villi, where nutrient absorption occurs, and crypts, which create a niche for pluripotent stem cells (Figure 1) [6,7]. Differentiated cells actively participate either in absorbing nutrients (primarily enterocytes) or as secretory cells. Goblet, enteroendocrine and tuft cells are located on the villi while Paneth cells are at the crypt bottom. Paneth cells intercalate between stem cells and contribute to the intestinal stem cell niche, which is crucial for ensuring the renewal process of the intestinal epithelium [4]. The outer layer of the intestinal epithelium is supported by the underlying stroma, encompassing mesenchymal cells, neurons and vasculature [6].

An ideal GI tract model should at least partially resemble this complex structure and allow interactions between the cells [8,9]. It should also provide in-depth analyses of host–gut microbiota interactions and deepen our understanding of their biological significance. It would be highly valuable if these models could generate high-throughput data using patient-derived microbiome samples [3].

## 2. Methods

A literature search was performed in PubMed (https://pubmed.ncbi.nlm.nih.gov/) between November 2023 and June 2024 using the keywords “bioreactor”, “in vitro model”, “in vitro fermentation”, “human gut”, “CRC”, “colorectal cancer”, “gastric cancer”, “intestinal model”, “oralization”, “microbiome”, “gastrointestinal”, “chemotherapy”, “constipation” and “diarrhea” in various combinations. Reference lists from the retrieved publications were also checked for additional relevant content.

## 3. Results

### 3.1. In Vitro Models of Gastrointestinal Tract

Several groups of in vitro models can be used to modulate the human GI tract. Simple models are based on cell culture, encompassing various 2D and 3D cell culture models, collectively called cell culture-based in vitro models. Alternatively, more advanced bioreactor-based models can be utilised, incorporating host cells or solely the gut microbiome for GI tract modulation. This chapter will explore the advantages and disadvantages of these models and their applications.

#### 3.1.1. Cell Culture-Based In Vitro Models

Cell cultures are commonly used to study cellular behaviour observed in vivo. The biochemical and biomechanical microenvironment intricately influences various processes, including cell differentiation, migration and growth [10]. Understanding the mechanisms driving these behaviours is crucial in the context of tumour development.

2D Engineered Gastrointestinal Tissue Models

The usage of intestinal epithelial cells in monolayer cultures remains common due to their ability to resemble the parent tissue phenotype [11]. Short-term monolayer cultures of intestinal epithelial cells have proven useful for assessing the impact of growth factors and morphogens on the behaviour of these cells [11]. Additionally, the techniques and instruments commonly used in standard laboratories are more readily available, as most of the imaging and manipulation methodologies were developed for 2D cultures [4]. Their availability in large quantities and their relatively stable phenotypes, transcriptomes and functional characteristics represent obvious advantages [12]. This simplified in vitro modelling system can be highly valuable for studying distinct aspects of disease mechanisms or the impact of various substances (e.g., anti-cancer drugs and bacterial metabolites) on the host cells. Epithelial cells in GI tract models come from primary intestinal cells or cell lines [8,9].

- Primary Cells

Primary cells are derived from patient tissues and processed for cell culture [13]. In 2014, researchers developed an enhanced human GI epithelial culture system. They isolated crypts from the biopsies obtained during routine endoscopy procedures to create 65 epithelial cell lines cultured in conditional media to form spheroids. Cell lines were generated from 47 individuals, including 25 patients with inflammatory bowel disease. These cells formed spheroids, which were grown on Transwell^®^ membranes to establish functional, polarised monolayers covered by a secreted mucus layer. The study also identified various adherence patterns with different strains of pathogenic *Escherichia coli* [14]. The advantages of this system involve using endoscopic biopsy tissue as the initial material and quickly expanding the spheroids. This allows for establishing a line from an individual patient within a timeframe that aligns with patient care, approximately 2–3 weeks. Another advantage of this novel spheroid culture system is that the protocol is relatively simple. The same protocol can be used to establish and maintain spheroid lines from any accessible GI site and there is no requirement for cell sorting.

- Immortalised Cells

However, despite these benefits, in monolayer cultures, cells lose their ability to self-renew and eventually undergo terminal differentiation. Thus, immortalised cells are used more for epithelium studies [15]. Immortal cell lines are widely used due to their easy handling, repeatability, availability and cost-effectiveness [9]. Cell lines, such as Caco-2, HT29, T84 and HT-29-MTX, were initially sourced from tumours [9,16]. The Caco-2 cell line has been the primary choice over the past two decades, recognised as the standard for predicting intestinal drug permeability [17]. It is a human cell line derived from colorectal adenocarcinoma and is commonly employed in models of the intestinal epithelium. In culture, these cells form a confluent monolayer and undergo differentiation, displaying behaviour similar to enterocytes [18]. Caco-2 cell differentiation initiates approximately 7 days after seeding, when the cells reach confluence, and typically concludes within 21 days. When fully grown, Caco-2 cells undergo polarisation, developing microvilli structures and exhibiting expression of several enzymes, transporters and receptors on the apical side. Tight junctions are formed between adjacent cells, enhancing the model’s accuracy in replicating the intestinal barrier [19,20]. However, the Caco-2 monoculture lacks important factors that influence enterocyte function, such as the presence of the mucus layer and interactions between the epithelium and the stroma [21].

To overcome these drawbacks, a cell model that consists of a co-culture of Caco-2 cells and mucus-secreting HT29 cells was developed. It is more advanced, as it mimics both enterocytes and goblet cells. HT29 cells are derived from human colonic adenocarcinoma cells, are resistant to exposure to methotrexate (MTX) and induce the differentiation of HT29 cells into mucus-producing goblet-like cells (HT29-MTX) [22]. Including mucus has been shown to enhance the predictability of experimental results, as observed across multiple studies and outlined in a recent paper [17]. An advancement in this co-culture model involved the addition of M-cell-like cells by combining Caco-2, HT29-MTX and Raji B cells. This combination induced the M-cell phenotype, enhancing the model’s physiological relevance [19,23].

T84, another frequently used cell line for studying the intestinal barrier, originates from a metastatic site in the lung derived from colon adenocarcinoma. Similar to Caco-2, T84 naturally differentiates into an enterocyte-like phenotype when fully grown, exhibiting polarity with microvilli and tight junctions, and desmosomes between neighbouring cells [24]. Also, the benefit of this cell line is the production of mucin, which it secretes in the culture [25]. In research concerning the physiology of the GI tract, especially regarding the impacts of microbial infection, T84 cells have been highly valuable. Their ability to form monolayers with high electrical resistance enables the detection of significant changes under experimental conditions [26].

- Stem Cell-Based Sources for the Gastrointestinal Tract Tissue Models

Mesenchymal stem cells (MSCs) are another source of cells for GI bioengineering and can be derived from bone marrow [27,28]. These cells can differentiate into epithelial [29] and smooth muscle cells, and act as a cell source for tissue engineering in the small intestine [30]. Another type of stem cell that can be used for disease modelling is cancer stem cells (CSCs). These are the cells within a tumour that can initiate tumour growth [31]. For example, a study by Dalerba and colleagues provided a robust and reproducible surface marker profile for CSC isolation and the possibility of modelling colorectal cancer (CRC) and metastatic CRC [31].

However, 2D cell culture models come with significant limitations. For instance, they typically involve single layers of cells and lack the diversity of cell types present in actual tissues, leading to the absence of crucial interactions between different types of cells. Moreover, the conditions in which they are cultivated may not faithfully replicate the natural environment, as serum in the culture medium introduces unknown variables [26]. Thus, there is a growing interest in employing interdisciplinary approaches that combine tissue engineering and microfabrication techniques to create more relevant and accurate tissue models [8].

- The Transwell^®^ System

To address the limitations of 2D cultures, the previously mentioned Transwell^®^ system was developed. It is a widely employed platform for studying intestinal permeability and the gut barrier in vitro. It involves seeding epithelial cells onto a membrane within an insert. This insert divides the system into apical and basal compartments, representing the intestinal lumen and blood vessels, respectively. Thus, it offers a mimicry of the in vivo gut barrier [32]. However, while these Transwell^®^-based models are an improvement over 2D cultures, they still lack the complexity of in vivo systems. These limitations have prompted the development of 3D models to mimic better the heterogeneous 3D environments regulating cell behaviour in vivo [17]. 

#### 3.1.2. 3D-Engineered Gastrointestinal Tissue Models

- Gastrointestinal Organoids

An organoid is a self-organising 3D structure cultivated in vitro, comprising organ-specific cells and demonstrating organ-like functions [4]. Primary intestinal epithelial cells can be cultured as self-organising epithelial organoid units [33] or as mixed cultures of epithelial and mesenchymal cells [34]. Generated from single stem cells, embryonic stem cells or induced pluripotent stem cells (iPSCs), these organoids have the capability for long-term culture, forming spherical or budding structures. These models provide near-physiological systems for diverse applications [4].

Tissue-specific adaptation techniques have facilitated the generation of organoids from nearly all parts of the GI tract, spanning from the small intestine and colon to the stomach, gallbladder, oesophagus, liver, pancreas, salivary glands and even taste buds [35]. In 2009, a long-term culture was developed where intestinal crypt structures were formed by supplementing Matrigel^®^ with various growth factors. This system facilitated the generation of crypt and villi organoids not only from crypts but also from stem cells, even without a non-epithelial cellular niche [33]. Most importantly, organoids can also be co-cultured with microorganisms, offering insights into pathogenesis and host–microbe interactions [36]. Before 2014, the culture of normal human gastric primary epithelial cells for extended experimental investigations was not feasible. However, a study by Schlaermann et al. introduced gastric organoids derived from normal human corpus mucosa. These organoids served as an advanced cell culture model, enabling in vitro studies on *Helicobacter pylori* infection [37]. Another study demonstrated that organoids develop a sealed lumen containing concentrations of α-defensins, effectively inhibiting the growth of various strains of *Salmonella enterica* for at least 20 h post-infection [38]. Likewise, experiments involving injecting bacteria like *H. pylori* into the lumens of gastric organoids have triggered robust inflammatory reactions in gastric gland cells [36]. Furthermore, bacterial components have been observed to show long-term effects on organoids from intestinal epithelial cells. For instance, Hibiya et al. noted decreased differentiation and increased NF-κB signalling in organoids even 11 weeks after 60-week exposure to a mixture of cytokines and bacterial cell wall components [39]. These advancements underscore how organoid cultures serve as excellent models for studying various diseases. In addition, organoids have been utilised to investigate the microbiome’s influence on CRC development. When microinjected into organoids, certain microorganisms have been shown to exacerbate cancer progression [40]. Furthermore, numerous studies have focused on establishing “Living Organoid Biobanks” by collecting organoids derived from diverse GI tract tissues and diseases, facilitating a range of applications including personalised and regenerative medicine, drug discovery and testing, biomarker development and disease modelling [41,42].

- Advanced 3D Intestinal Models

Advanced 3D intestinal models feature multi-layered organisation with cells embedded in ECM-like substrates like Matrigel^®^ or hydrogels. An example includes a 3D co-culture model of human intestinal epithelial cells with immunocompetent macrophages and dendritic cells, successfully replicating an elevated inflammatory cytokine response [43]. However, it is crucial to note that despite their 3D structure, these models do not feature the characteristic crypt and villi architecture. Thus, biofabricated scaffolds were created to imitate the gut’s villi and crypt architecture or even tubular structure [17]. In this case, careful consideration of the scaffold’s material characteristics had to be taken [44]. Scaffolds can consist of various materials, such as polyvinyl alcohol/gelatin; thiol–norbornene; natural polymers like nanofibrillar cellulose, alginate, collagen and chitosan; and synthetic polymers like peptide nanofibre scaffold and decellularised matrix such as porcine liver-tissue derived extracellular matrix [45]. When cultured on synthetic and alginate hydrogel scaffolds, Caco-2 cells displayed differing responses. In the study by Dosh et al., cells showed enhanced viability on synthetic hydrogel scaffolds, whereas alginate scaffolds prompted spheroid formation. Conversely, synthetic hydrogel scaffolds facilitated the development of villi-like structures [46]. Another study demonstrated the capability of silk fibroin scaffolds to support the attachment, proliferation and differentiation of human gastrointestinal epithelial and smooth muscle cell lines. It was shown that silk fibroin scaffolds supported notably higher attachment levels for small intestinal smooth muscle cells, colon smooth muscle cells and oesophageal smooth muscle cells than small intestinal submucosa [47].

These models provide an enhanced understanding of cell-to-cell interactions and the pathophysiology of GI diseases and hold the promise of providing targeted and personalised treatments for individuals with GI dysfunction [48]. Three-dimensional systems have been used for a long time in cancer research for their safety and efficacy in testing and research. The current applications of 3D cell culture in therapeutics and diagnostics are reviewed in [49]. Developing dynamic 4D systems was one of the achievements made possible by 3D cell cultures. Han and colleagues created a hydrogel that responded to various stimuli, such as temperature, small molecules and enzymes, by forming macropores [50]. This method showed potential applications in studying how changes in surface features affect cells and as a means for delivering cells. The future of 4D systems will depend on further progress in material systems that have adjustable structures and responsive signalling [51].

Considering the significance of the gut microbiome on GI cancer development and progression [52,53], it would be advantageous to incorporate it as a crucial variable in GI tract disease modelling. The next section of this chapter will examine the potential for GI tract modulation using bioreactor systems.

### 3.2. Bioreactor-Based In Vitro Models

Technological advancements play a vital role in understanding the diversity and functions of the gut microbiota. Bioreactor-based in vitro gut fermentation models offer valuable insights into the composition and functionality of the microbiome derived from the stool samples while reducing animal testing and overcoming the lack of complexity of cell culture-based models.

The standard bioreactor design is a glass, carbonate or stainless steel cylinder with a flat bottom, which maximises the vessel area or dished/hemispherical bottom, as that shape can handle a higher pressure [54]. The vessel contains an impeller driven by an overhead motor, essential for mixing the components. Typically, only 75–80% of the volume of stirred bioreactors is filled with liquid to allow for the disengagement of droplets from the exhaust gas and to accommodate any foam [55].

Microbiome cultivation success is highly dependent on the stool sampling procedure. The simplest method is when a donor collects their stool in a special container, which helps prevent sample contamination and ensures the right conditions for transportation. The main advantages of this method for donors are simplicity, affordability and non-invasiveness. For the researchers, it is advantageous, as there are guidelines and protocols for stool collection, transportation and subsequent procedures [56]. Another method to collect microbiome samples is the aspiration of intestinal fluid. This could be achieved through capsules, tubes or endoscopic aspiration; moreover, this method prevents contamination as the sample is isolated from the external environment after collection. However, it is technically challenging, time-consuming and invasive, and the donor cannot perform the sampling procedure without assistance [57]. In the future, samples could be collected by swallowing a capsule, which moves through the GI tract by peristalsis. The main advantages are low invasiveness, accurate location of sampling points and high technological content (e.g., locomotion mechanisms, wireless connection, temperature, pH, pressure, oxygenation, oxidation/reduction conductivity sensors and multi-axial accelerometers and gyroscopes for inertial navigation and positioning). However, there are drawbacks, such as high cost, the risk of capsule aspiration or retention [57] and possible sample contamination by intestinal fluid from non-collected sites [58].

The in vitro models aim to mimic the GI environment’s biological conditions to support the microorganisms’ optimal growth. Stability in the culture is achieved by controlling process parameters and maintaining constant environmental conditions throughout the culture period [57]. In vitro models typically are categorised into the following two groups: (i) batch fermentation and (ii) continuous fermentation models, both designed to simulate the human GI tract [59].

In this review, we have chosen to depart from this categorisation and instead organise the models based on the number of vessels and the level of throughput. Each model has advantages and disadvantages, so the choice of model will ultimately depend on the study’s specific objectives. An extensive summary is provided in Supplementary Table S1.

#### 3.2.1. Single-Vessel In Vitro Models

- Batch Fermenter

**Figure 2:**
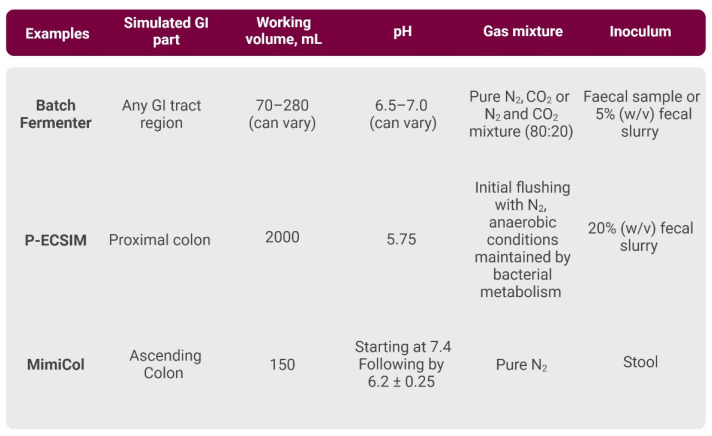
Brief overview of the primary characteristics of single-vessel bioreactors. Supplementary Table S1 contains a more comprehensive description and relevant references. Created with BioRender.com.

The batch fermenter represents one of the earliest and most basic static in vitro systems (Figure 2). Batch operation is a closed process in a vessel, i.e., there is no inflow or outflow of the reactor during the run. In this fermenter, all the components are added at the beginning of the fermentation process in a fixed volume (typically ranging between 70 and 280 mL [60,61]), and nutrients or substrates are not added during the process. During the run, nutrient consumption increases due to increasing cell concentration. Cells are in the exponential phase, growing until the nutrients are used. Once nutrients are used, cell death occurs, leading to zero growth rate at the end. The experimental conditions include maintaining a temperature of 37 °C, a pH regulation between 6.5 and 7, continuous stirring and sustaining anaerobic conditions through nitrogen supply [60,61]. Anaerobic conditions are achieved via pure nitrogen (N_2_) flushing or a gas mixture of carbon dioxide (CO_2_) and hydrogen (H_2_). With this uncomplicated experimental configuration, in vitro studies of the human GI tract can be conducted within a timeframe of 24–48 h [60,61].

Until today, many studies continue to utilise batch bioreactors due to their affordability, ease of use and rapid setup [62]. However, these systems come with significant drawbacks. They cannot be easily integrated with other systems [57] and only simulate a single GI tract zone, limiting their ability to replicate more intricate GI tract functions [63], and are better suited for short-term cultures [64]. To overcome shorter culture time, a closed fed-batch mode was developed. Initially, the fed-batch mode of operation starts as a batch phase where nutrients decrease and cells grow exponentially. Before the nutrients become a limiting factor, media is added at a consumption rate throughout the remaining fermentation duration, thus prolonging operation time. The rate of medium addition determines the growth rate. Conditions in the bioreactor are constant except for the growing biomass and waste product concentrations. This mode is more complex than batch mode due to the addition of the medium. In addition, no waste products are removed from the bioreactor and, therefore, the reaction volume changes over time [65].

- P-ECSIM

The Proximal Environmental Control System for Intestinal Microbiota (P-ECSIM) is an in vitro system consisting of one bioreactor, mimicking the proximal part of the large intestine. It has a working volume of 2 L and operates in a 24-h batch operation mode followed by continuous cultivation. The temperature is kept at 37 °C and the pH is maintained at 5.75 by adding 2 M NaOH automatically. The stirring speed is set to 400 rpm. The gassing with N_2_ occurs immediately before the run and anaerobic conditions are ensured through the self-maintenance of metabolic bacteria. The retention time has been set and used in different experiments, allowing two different variations: a shorter time of 12.5 h and a longer version of 25 h [66]. P-ECSIM has mainly been used to research bacterial metabolism and the impact of transit time on microbial variation [66,67].

- MimiCol

The Mimicking Colonic Microbiota model (MimiCol) simulates the ascending colon. The experimental volume of the reactor is 150 mL. Temperature of 37 °C is maintained by water bath and pH is set at 6.2 ± 0.25 with a starting value of 7.4. pH of 6.2 is reached within the first two hours. Mechanical motions and homogeneous mixing are reached using two perpendicular blades with an agitation rate of 6 rpm. Anaerobic conditions are achieved by the constant flushing of N_2_ through the headspace of the reactor. Until now the MimiCol model has been used to study the dissimilation of the drug sulfasalazine [68], but it has ample potential to be implemented in other fields as well.

#### 3.2.2. Multi-Vessel In Vitro Models

**Figure 3:**
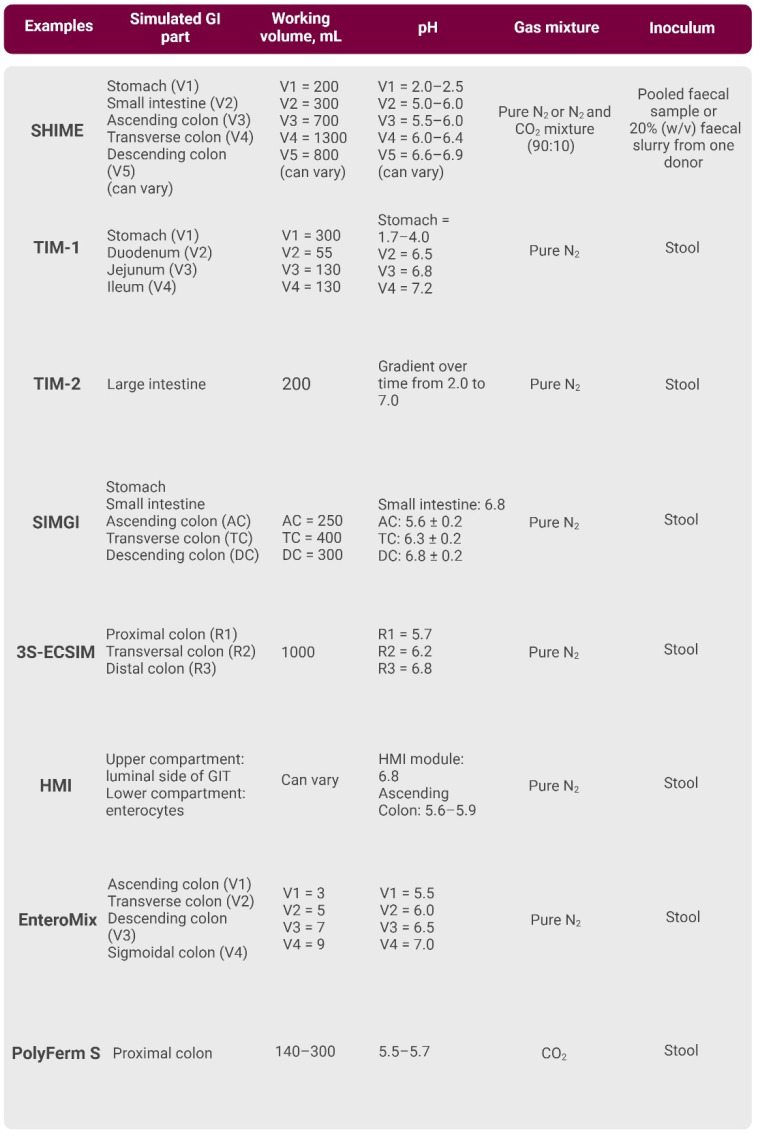
Brief overview of the primary characteristics of multi-vessel bioreactors. Supplementary Table S1 contains a more comprehensive description and relevant references. Created with BioRender.com.

Due to the different environmental conditions in the colon region of the human GI tract, one-vessel models rapidly reach their limit. This is because the simulation of dynamic changes in parameters like pH value, nutritional content or microbial diversity is difficult. Nevertheless, to simulate the different parts of the GI tract, multi-vessel models consisting of multiple reactors have been invented [69,70,71]. One of the first models that simulate proximal, transversal and distal colon conditions was created at the University of Reading (Reading, UK) and presented by Gibson et al. [72]. This model consists of three increasing-sized vessels, mimicking the proximal, transverse and distal colon conditions. The operating conditions include setting the pH of the vessels at 5.5, 6.2 and 6.8, respectively. The first vessel represents a rapid bacterial growth rate and is operated at an acidic pH, like the proximal colon. The third vessel resembles neutral pH, slow bacterial proliferation rate and low substrate availability, which resembles more distal regions of the human GI tract [71]. This part of the publication will explain the general setup of the multi-vessel model and compare some of the most commonly used in vitro multi-vessel modulation systems (Figure 3).

- SHIME

The Simulator of the Human Intestinal Microbial Ecosystem (SHIME) system consists of double-jacketed glass vessels connected through peristaltic pumps. Fresh medium is added three times per day in the gastric compartment and pancreatic and bile liquid is added to the small intestine compartment [73]. During digestion in these three compartments, the slurry is pumped into the ascending colon vessel where digestion is continued. This system is usually inoculated with a faecal microbiome from one donor [74]. Vessels are inoculated with 50 mL of a 20% faecal suspension and prepared in an anaerobic sodium phosphate buffer (0.1 M, pH 7.0) [75]. The liquid in these three vessels is continuously stirred with magnetic stirrers at 150 rpm. This system controls all necessary parameters, such as pH, temperature, nutrient supply, transit time, enzyme and bile acid concentration, and the anaerobic atmosphere [73]. The pH in gastric vessels is fixed at a pH of 2.0 and the pH of the colon compartments is controlled between 5.6 and 5.9 in the ascending, 6.1 and 6.4 in the transverse and 6.6 and 6.9 in the descending colons. The pH is controlled by 0.1 M HCl and/or 0.1 M NaOH [75]. All vessels are kept under anaerobic conditions by flushing the headspace of the vessels with N_2_ or 90%/10% N_2_/CO_2_ mixture [74] twice a day for 15 min. [75]. The environmental conditions in all vessels are entirely computer-controlled.

The experiment using SHIME consists of four different phases [74] after inoculation and the microbial community adapts to its environmental conditions (two weeks). Then, the reactors are operated at nominal conditions, and baseline parameters are measured (two weeks). Thereafter, specific treatment of the microbial community takes place (2–4 weeks). Finally, the determination period follows, where the time course of changes induced by the test substance is determined (2 weeks).

The SHIME system provides highly reproducible results because physical parameters, such as temperature, levels of dissolved oxygen and pH, are controlled by the operator. In 2010 and 2013, Van den Abbeele et al. investigated the microbial colonisation process with a high-resolution phylogenetic microarray using two identical SHIME systems. According to the results, microbial community composition reached a steady state after 2 weeks and functional stability after 3 weeks. This colonisation process was reproducible in both SHIME units and resulted in highly diverse microbial communities. Thus, if it is impossible to run SHIME experiments simultaneously, then this system still provides a reproducible microbial colonisation process and metabolic activity [76,77].

In the past 30 years, since the introduction of the multi-vessel model SHIME [73], it has been improved by several research groups [76,78,79]. The TWIN-SHIME comprises five bioreactors (stomach, small intestine, ascending colon, transverse colon, descending colon), simulating the entire GI tract. The vessels are inoculated with the same faecal sample and the SHIME units are run under the same environmental factors. The microbial stabilisation time is reached after two weeks [76]. Another variation of the SHIME system is the M-SHIME (mucosal-SHIME) model. Therefore, scientists can study the mucosal community by adding a mucus layer. This M-SHIME model can further be combined with the TWIN-SHIME [76].

- TIM-1 and TIM-2

Minekus et al. have developed a multi-compartmental model referred to as the TNO Gastro-intestinal Model (TIM). This model comprises two main components: TIM-1, which simulates the stomach, duodenum, jejunum and ileum, and TIM-2, which simulates the large intestine [80,81]. The development of this model was driven by the industrial need to investigate food products under physiological conditions. It integrates the precision and consistency of a model system with physiological parameters, including mixing, transit of meals in the colon, varying pH levels, secretion and composition of digestive fluids and elimination of waste products. Through computer-controlled simulation, this physical model emulates the fundamental in vivo parameters [82].

The TIM-1 model represents the most utilised configuration within the TIM series. Various compartments are equipped with peristaltic valve pumps, facilitating the transfer of liquids from one compartment to another. Mixing is accomplished through its flexible inner walls, where water is circulated into the space between compartments to maintain a constant temperature of 37 °C. Anaerobic conditions are maintained by flushing vessels with N_2_ [82]. The pH levels are controlled with sodium bicarbonate according to the pH curve, where in the stomach compartment, pH is set from 4.5 to 1.7, at 5 to 90 min. In the small intestine compartments, pH 6.5, 6.8 and 7.2 are maintained for the duodenum, jejunum and ileum, respectively. The pH of the large intestine vessel is maintained at 5.8, controlled by the addition of 1 M NaOH, to neutralise acids produced by the microbiota [75,83]. To prevent microbial death resulting from the build-up of microbial metabolites, the model employs a waste removal strategy tailored for water-soluble and lipophilic by-products. Water-soluble waste products are eliminated using dialysate, a 5 kDa dialysis membrane, and a container to collect the spent dialysate. This dialysis system is advantageous as it enables the maintenance of an active microbiome for a period of up to 3 weeks [83]. The current system is unable to eliminate lipophilic waste products, as they are encapsulated within micelles that are too large to traverse the dialysis membrane. Consequently, the removal of lipophilic products necessitates the use of a 50 nm filter [82]. Usually, experiments with the TIM system are performed over a period of one week [83].

The TIM-2 model consists of four interconnected glass vessels with a flexible membrane inside. Double-jacketed glass vessels and temperature sensors allow control of the temperature. In the TIM-2 model, the application of regular pressure on water at specific intervals and in a particular sequence causes the membrane to contract, simulating peristaltic movements. This system differs from the TIM-1 model in that it does not involve the phase separation of solids and liquids as observed in other systems. Additionally, it utilises a 50 kDa hollow-fibre membrane, a feature previously discussed in this review [80]. The model is inoculated using a faecal sample in two distinct ways. The first approach involves introducing a faecal sample from one individual into one of the TIM-2 systems, whereas the donation from a second individual is inoculated into the second unit of the system. Subsequently, the composition of the microbiota can be compared. The second approach entails pooling faecal samples from multiple donors to create a standardised microbiota suitable for various experiments. This approach facilitates the comparison of different conditions, all starting with the same microbiota composition [83]. After inoculation, the microbiota is allowed to adapt over a period of 16 h. The microbiota in this system is fed through a food syringe containing a simulated ileal efflux medium (SIEM), which in composition mimics the components that reach the colon from the terminal ileum through the ileal–cecal valve [83]. After the adaptation period, there is a 2–4 h starvation period to allow fermentation of all available carbohydrates in this system. After starvation, the medium is replaced by a carbohydrate-rich medium and fed for 72 h [83]. The TIM system offers the advantage of rapid experimentation, typically completed within one week. This system replicates peristalsis and incorporates a dialysis procedure, enabling the maintenance of normal microbiota density and the production of metabolites at physiological concentrations.

The TIM system has various adaptations, such as a simplified version called TinyTIM, designed for studies that do not require separate intestinal steps. Another improvement is TIM-age, which is a single small intestinal compartment with a flexible wall that gradually contracts and consequently removes volume to better mimic digestion in the stomach [82].

- SIMGI

One of the novel in vitro models is called the Simulator of the Gastrointestinal Tract (SIMGI) [84]. This model consists of five vessels that simulate the stomach, the small and the large intestine (ascending, transverse and descending colon) using a computational automated control system. The ascending colon is filled with 250 mL, the transverse colon with 400 mL and the descending colon with 300 mL of nutritive medium [85]. The total residence time is 76 h and modified from the standard SHIME system [73,76,79,84]. The temperature is settled at 37 °C and the pH is adjusted as follows: intestine 6.8, ascending colon 5.6 ± 0.2, transversal colon 6.3 ± 0.2 and descending colon 6.8 ± 0.2. The content in the stomach is mixed by peristaltic motions caused by the pumps of the water jacket in the fermenter. Each reactor of the small and large intestine is blended at 150 rpm continually using magnetic stirrers. Additionally, N_2_ is supplied constantly through a port [84]. Regarding research applications, the SIMGI model is mainly used to explore diet-related diseases [71]. Moreover, this model is used to study the potential effects of prebiotics [86].

- 3S-ECSIM

The Three-stage Environmental Control System for Intestinal Microbiota (3S-ECSIM) model is derived from P-ESCIM [66]. While P-ECSIM only simulates the proximal colon, 3S-ECSIM is advantageous as it mimics the distal and transversal parts of the colon [87]. The system consists of three vessels, mimicking the proximal, transversal and distal colons [67]. The volume of the bioreactors, operation mode, temperature and gassing are the same as described for the P-ECSIM model [67,87]. The pH is settled as follows: proximal colon 5.7, transversal colon 6.2 and distal colon 6.8. The agitation rate is set at 400 rpm [87] using a marine propeller and Rushton turbine [67]. The microbial stabilisation phase persisted for 240 h and the retention time lasted 12.5 h in the proximal colon and 17.7 h in the transversal and distal colons. The medium exchange between the vessels is enabled using peristaltic pumps; additionally, the first reactor is constantly supplied through peristaltic pumping [87].

- The Host–Microbiota Interaction Model

In 2014, the host–microbiota interaction (HMI) model was introduced to study the indirect host–microbiome interaction in the GI tract [88]. This module simulates the processes occurring at the level of the gut wall, including shear stress, metabolites and gas permeation, bacterial adhesion and host response. It consists of two parallel vessels that allow it to perform experiments in duplicates. Each setup consists of two compartments where the upper compartment mimics the luminal side of the GI tract and the lower compartment simulates the host by having host cells, such as enterocytes. The functional double layer, which separates the compartments, is composed of a mucus layer and a semi-permeable polyamide membrane. The mucus layer thickness ranges from 200 to 250 μm. The polyamide membrane has a thickness of 115 μm and a pore size of 0.2 μm. This double-membrane structure provides a mucosal area, which is colonised by gut bacteria. In addition, it allows the transport of low-molecular-weight metabolites and oxygen and protects the host’s cells from the toxic effect of direct contact with the microbial community [88].

The HMI module was used in combination with other GI tract modelling systems. Two HMI modules were connected to the ascending colon vessel of SHIME where the last vessel mimics the ascending colon. The system’s effectiveness was evaluated by three distinct molecular methods: quantitative polymerase chain reaction, denaturing gradient gel electrophoresis and fluorescent in situ hybridisation. All in all, molecular data showed that by connecting the HMI module to the SHIME, it was possible to evaluate the modulating effect of the test product on both the luminal- and mucosa-associated microbiota. And, compared to other systems, it is advantageous, as it allows for the simulation of bacterial adhesion to the gut and the indirect effect on human cell lines [88].

- The EnteroMix Model

The EnteroMix model consists of four glass vessels with four parallel units, representing the ascending, transverse, descending and distal colons. The vessels have very small working volumes of 12 mL maximum (usually 3, 5, 7 or 9 mL). The pH of the system is fully automated and is 5.5, 6.0, 6.5 and 7.0 for the individual vessels, respectively [89]. The temperature in this system is not controlled by water jackets. Thus, it is maintained at a thermostatic room temperature of 37 °C. The constant supply of N_2_ maintains anaerobic conditions.

Using this model, it is possible to run four simulations using the same faecal inoculum. The inoculum is mixed in the first vessel and 10 mL of the mixed culture is pumped into the next vessel. After 3 h of incubation, fresh medium is presented to the first vessel. The fermentation and fluid and fresh medium exchange continues for 48 h, after which the process stops and sand samples are collected from each digestion vessel [88]. The final volumes of the vessels are 6, 8, 10 and 12 mL, respectively.

- The Polyfermentor Intestinal Model

In 2013, a novel Polyfermentor Intestinal Model (PolyFermS) was presented, aiming to overcome issues of reproducibility and biological replication by entrapping the faecal microbiota in gel beads [90]. PolyFermS consists of vessels containing 140 mL of medium with controlled pH and anaerobic conditions. This model differs from other systems because it uses faecal samples from healthy individuals immobilised in gel beads of gellan gum, xanthan gum and sodium citrate. These beads are colonised for 48 h in batch cultures under gut-like conditions: 37 °C, pH 5.7, controlled by 2.5 M NaOH and continuous flow of CO_2_ in the bioreactor [89]. Liquid of the first-stage continuous inoculum reactor containing immobilised faecal microbiota and mimicking the proximal colon is used to continuously feed a set of second-stage control and test reactors, operated in parallel with conditions of the proximal colon [90].

#### 3.2.3. High-Throughput In Vitro Models

- MimiCol^3^

**Figure 4:**
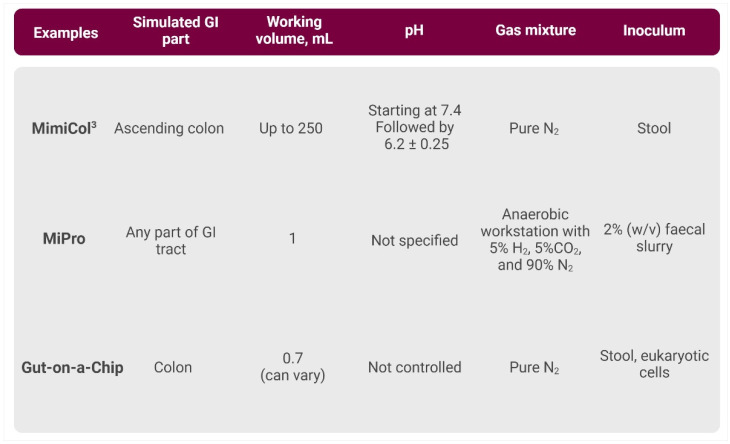
Brief overview of the primary characteristics of high-throughput systems. Supplementary Table S1 contains a more comprehensive description and relevant references. Created with BioRender.com.

MimiCol^3^ is one of the recently improved high-throughput models of the human gut microbiome (Figure 4). This model has been further developed from the first version of MimiCol, both imitating the ascending colon [68,91]. One of the main aims of the operating research group was to design a simple setup that can be up-scaled easily. Relative to the first version of MimiCol, the advantage of MimiCol^3^ is that it performs three parallel experiments of the ascending colon using the same conditions. This allows the collection of more dependable data in a shorter time, which makes it a so-called “high-throughput model” [68].

MimiCol^3^ operates under the same temperature regime and pH value and is also gassed continuously with N_2_ [68,91]. A pH electrode monitors the pH value and peristaltic pumps deliver 1 M HCl and 1 M NaOH to maintain the specified pH value. The agitation rate is set at 100 rpm using trembling motions [68].

As the MimiCol^3^ model is newer, just a few research studies are available. In particular, the investigation of the impact of the drug sulfasalazine has already been explored [68,92].

- 96-Well-Plate-Based Models

In 2018, Parrish et al. introduced the 96-well microplate bioreactor platform, which offers experimental flexibility. This model was used to modulate ovarian cancer and expose it to the anti-cancer drug doxorubicin [93].

Another possibility of a high-throughput model using a simple laboratory setup was shown by Li et al. [94]. MiPro model is a 96-deep-well-plate-based culturing model that maintains an individual’s gut microbiome’s functional and compositional profiles. The 96-deep-well plate is covered with a silicone gel cover perforated at the top of each well. This cover impairs gas exchange with the outer environment in the anaerobic chamber to preserve the partial pressure of gases and volatile metabolites in each well, which could subsequently preserve certain levels of dissolved gas molecules in the culture medium. Mixing of the medium is ensured simply by putting this plate on a shaker inside the anaerobic chamber. As proposed in the publication, this in vitro gut microbiome model is used for scalable investigation of drug–microbiome interactions, such as during high-throughput drug screening [94].

- Gut-on-a-Chip Models

As previously mentioned, the 3D culture system offers a significant advantage over 2D monolayer cultures by providing a more physiologically relevant tissue organisation. Organ-on-a-chip technology has enabled new ways to study organ-level functions and provides an alternative technology for complementing studies on health, tissue development and disease [95].

The gut on a chip is a 3D microfluidic device that mimics the dynamic environment of the human intestine [96]. These small chips consist of two microfluidic channels divided by a flexible and permeable membrane. These channels stand for the luminal part of the gut, which includes gut epithelial cells, and the second channel is shown to represent the vascular channel, which includes vascular endothelial cells. The chips are permeable, allowing gases like CO_2_ and O_2_ to pass through. Due to the application of vacuum, this membrane can stretch and contract and, therefore, simulate breathing processes [96].

In 2016, Esch et al. introduced a modular, pump-less body-on-a-chip platform for the co-culture of GI tract epithelium and 3D primary liver tissue. It was demonstrated that cells maintained their viability for up to two weeks, with liver cells exhibiting low cell death rates [97]. Chen et al. demonstrated a similar approach in 2018, where primary GI epithelial cells from intestinal biopsies were cultured with human hepatocellular carcinoma cell line HepG2 C3A in a single-organ fluidic culture system. This study showed the potential to sustain normal physiological responses and maintain cell viability for up to one month [98]. In 2022, a new protocol was published, outlining a five-day methodology to regenerate functional intestinal microarchitecture using a gut-on-a-chip system or a hybrid chip with Transwell^®^ inserts. This approach mimicked GI conditions with relevant sheer stress and mechanical motions [99].

Another interesting approach that allows the co-culture of human and microbial cells under conditions representative of the gastrointestinal human–microbe interface is the HuMiX (human–microbial crosstalk) [100]. A more detailed description of this model and other gut-on-a-chip platforms can be found in a recent review [101]. Additionally, there was a new approach of 3D bioprints that allowed physiologically relevant studies of the interactions of host GI tract cells by culturing them with commensal and pathogenic bacteria under anaerobic conditions [102].

### 3.3. Bioreactor-Based Approaches to Study Gastrointestinal Tract Cancer

The lack of progress in finding effective cancer treatments may be due, in part, to the lack of accurate models that mimic pathological processes. Conventional 2D cell cultures have provided great insight into the ability of tumour cells to grow, but they do not provide information about the complex interactions between the cancer cells and the physicochemical environment within living tumours. For this reason, many groups have explored the use of 3D in vitro models, and, more recently, microfluidic devices have been applied for this purpose as well [103]. Most importantly, in vitro models can be customised to replicate specific aspects of GI diseases, focusing on one part or imitating different parts of the GI tract. This includes incorporating factors like inflammation, dysbiosis and altered host–microbe interactions to study disease mechanisms and test potential therapeutic interventions.

Several research groups utilised bioreactor-based in vitro systems to modulate CRC and tumour microenvironment (TME). Manfredonia et al. used a perfused bioreactor to investigate if this system can maintain the main TME cellular components in primary CRC samples [104]. Fragments from freshly resected tumours were placed in the bioreactors and cultured for 3 days using static or continuous operation mode with perfusion. It was found that static culture resulted in complete tissue loss while perfusion-based culture of primary CRC specimens resembled key features of TME [104]. A study by La Rocca et al. used spinner flask bioreactors to produce 3D in vitro CRC tumour microtissues to reproduce TME. This study included the CRC cell line HCT-116 and primary normal human dermal fibroblasts [105]. Microtissues were cultured for 12 days using semi-continuous operation mode with medium exchange every two days. The efficacy of developed microtissues as a drug-screening platform was evaluated by examining the impact of 5-FU, curcumin-loaded nanoemulsions and their combination. The newly developed microtissues have been observed to accurately replicate the TME by demonstrating extracellular matrix remodelling, cell proliferation and the induction of normal fibroblasts into an active phenotype. Furthermore, the authors state that these microtissues have the potential to be integrated with tissue-on-a-chip technologies to further investigate studies related to cancer progression and drug discovery [105]. These studies offer valuable insights into the development of the TME, which is a contributing factor in the progression of the disease.

An additional crucial consideration is the development of reliable and reproducible cancer models to facilitate the evaluation of various substances, including new anti-cancer drugs. The methodology employed by Hirt et al. involved the cultivation of CRC cells in porous scaffolds under perfusion flow to engineer tissue-like structures [12]. In this study, perfused 3D cultures demonstrated more uniform cell seeding and higher cell counts than static cultures. Transcriptome analysis further indicated a stronger correlation between xenografts and perfused 3D cultures. Notably, treatment with 5-FU triggered apoptosis, down-regulated anti-apoptotic genes and reduced cell numbers in 2D cultures, whereas it only induced “nucleolar stress” in perfused 3D cultures and xenografts [12]. In 2021, Gouws et al. demonstrated the significance of bioreactor-based models in drug testing. The study evaluated South American medicinal plants *Sutherlandia frutescens* and *Xysmalobium undulatum* for potential activity against CRC. The researchers employed clinostat-based rotating bioreactors operating in semi-continuous mode and utilised a 3D sodium alginate-encapsulated LS180 CRC spheroid model. This approach effectively assessed the anti-cancer potential of the plant extracts against CRC [106]. The Rotary Cell Culture Systems (RCCS) were employed to investigate the potential impact of altered gravity on cell viability, drug delivery efficacy and the modulation of treatment-resistance-related gene expression in the context of gastric cancer [107,108].

However, it is important to note that none of the cancer studies mentioned in this chapter used bioreactors to incorporate the complex gut microbiome from the stool as an inoculum. These studies only involved mammalian cells, tissues or a limited number of bacterial species, thereby overlooking the complexity of cell-bacterial interactions in the gut.

### 3.4. Modelling the Side Effects of Cancer Treatment

However, several research studies in the field of oncology have addressed this limitation by integrating the microbiome in bioreactors and utilising it as a faecal inoculum. These investigations encompassed the evaluation of adverse effects of cancer treatment, including radiation-induced toxicity, dysbiosis resulting from chemotherapy and antibiotic usage, and the oralisation process induced by prolonged proton pump inhibitor (PPI) use.

#### Radiation-Induced Toxicity

Radiotherapy is currently employed as a CRC treatment strategy, but due to the radiotoxicity, it has a significant impact on healthy tissues and can raise health concerns [109]. Many CRC survivors have undergone radiation therapy for tumours in the pelvis or abdomen, thus rendering the bowel at risk for injury. The current prevalence of patients with long-term radiation-induced intestinal side effects exceeds that of ulcerative colitis and Crohn’s disease combined [110]. A recently published review by Bogues et al. [3] focused on translational research for pelvic radiotherapy-induced toxicity. This was achieved by developing a humanised in vitro model that mimics radiotherapy treatment conditions and allows the assessment of radioprotective agents without animal testing.

- Chemotherapy and Antibiotics-Induced Dysbiosis

A study by Ichim et al. (2018) implemented a Triple-SHIME system to study chemotherapy and antibiotic-induced dysbiosis [111]. This study aimed to evaluate the bioactivity of a supplement consisting of capsules with a blend of probiotics of the genera Lactobacillus and Bifidobacterium plus 10 digestive enzymes in protecting the human GI tract from chemotherapy and an antibiotic. A faecal sample from a healthy adult donor was used to inoculate the system and the microbiome was co-cultured with Caco-2 and THP-1 cells. Interestingly, the probiotic with digestive enzymes supplemented fermentation activity in the colon reactors and accelerated the recovery of microbial populations following 5-FU/vancomycin treatment. In the proximal colon, preventative administration of the supplement resulted in full recovery of the gut microbial community after cessation of 5-FU and vancomycin treatment [111]. Perturbation of the colonic ecosystem by antibiotic therapy was also modulated using the ARCOL model and faecal samples from three healthy donors [112]. A simpler bioreactor system, consisting of the gas impermeable bottles sealed with butyl rubber caps and inoculated with the faecal microbiome from CRC patients, was used to study the association of colonic methane, formed by methanogenic archaea, and pH with gastrointestinal symptoms induced by adjuvant 5-FU chemotherapy. It was found that methane producers had less frequent diarrhoea during chemotherapy than non-producers and more frequent constipation. Baseline faecal pH was also associated with symptoms during chemotherapy: the higher the pH, the lower the risk of diarrhoea, and the higher the risk of constipation. This study also underscores the importance of the intestinal microbiome in the development of intestinal toxicity during 5-FU therapy [113]. Another study by Blaustein et al. (2021) developed an in vitro model to characterise the key changes in bacterial community dynamics under chemotherapeutic treatment and the role of bacterial interactions in drug detoxification to promote microbiome resilience. For this goal, batch fermentation was used, but no stool inoculum was used. Inoculation occurred with a special bacterial composition that had drug-sensitive strains. Bacteria with predicted resistance involving biotransformation significantly lowered concentrations of doxorubicin in culture media, permitting the growth of drug-sensitive strains in monoculture. Such protective effects were not produced by strains with drug resistance conferred solely by efflux. In the mixed communities, the resilience of drug-sensitive members depended on the presence and efficiency of transformers as well as drug exposure concentration [114].

- Oralisation Process

Another cancer treatment side effect could be an oralisation process, which can be induced by the long-term usage of PPIs [115,116]. The proof-of-concept study by Etienne-Mesmin (2023) proposed a new model of oral-to-gut invasion by the combined use of an in vitro model simulating both the physicochemical and microbial (lumen- and mucus-associated microbes) parameters of the human colon (M-ARCOL) [117]. This study used stool samples from two healthy donors to inoculate the bioreactor. Oral invasion of the intestinal microbiota was simulated by injection of enriched saliva in the in vitro colon model inoculated with a faecal sample from the same healthy adult donor. The mucosal compartment of M-ARCOL was able to retain the highest species richness levels over time, whereas species richness levels decreased in the luminal compartment. This new model of oralisation provided useful mechanistic insights into the role of oral microbiome in various disease processes. The authors of this paper developed a bioreactor-based in vitro model of the oralisation, which adapts the commercially available DASbox^®^ mini-bioreactor system and uses stool samples from healthy donors for the inoculation (K.Ž. manuscript in preparation). The benefit of this system compared to that previously mentioned is that it does not require saliva samples from donors as it implements previously confirmed oralisation biomarkers *Streptococcus salivarius* and *Veillonella parvula* [116], and allows a precise spiking strategy, which can be further used for microbiome–drug interaction studies.

## 4. Discussion

Human diseases are complex, involving interactions among different organ systems, microenvironments and immune responses. In contrast, in vitro systems can offer clarity and standardisation. Replicating the functionality observed in living organisms in an artificial environment is crucial for accurately simulating diseases, yet it presents a great challenge for researchers. Acknowledging that no single model can address all research questions is crucial. Each model offers distinct advantages that make it adaptable to specific uses. The wide variety of bioreactor-based technologies has facilitated the exploration of various aspects concerning GI tract diseases. This review specifically examined models for GI cancer as well as the adverse effects associated with cancer treatment, including radiation-induced toxicity, chemotherapy-induced dysbiosis and the oralisation process. This review outlined several applications of bioreactor-based models but did not encompass all potential uses. These applications may include the investigation of drug development, delivery and side effects [118,119], the modulation of different cancer tissues [120] as well as studies focusing on metabolic comorbidities [121].

Numerous research studies have consistently utilised batch bioreactors due to their cost-effectiveness, user-friendly operation and rapid setup [62]. These systems, however, have notable limitations as they are not readily compatible with other systems [57] and only simulate a single GI tract zone, which restricts their capacity to reproduce more complex GI tract functions and interactions [63]. Also, they are better suited for short-term cultures [64].

Thus, single-vessel models with different operational modes were developed. Comparing the single-vessel models P-ECSIM and MimiCol, both systems have the potential to replicate the proximal part of the large intestine. Primarily, both models are utilised for fermentative studies investigating bacterial metabolism [66,91]. The operational parameters of both systems are different as the working capacity of the 2 L P-ECSIM model exceeds the 150 mL volume of MimiCol. The smaller volume of MimiCol offers advantages in cost-effectiveness, requiring less material compared to the P-ECSIM [66,91]. Contrarily, the MimiCol model entails higher costs for continuous gassing [91], whereas the P-ECSIM method involves initially purging the reactor with N_2_, after which bacterial metabolism automatically sustains anoxic conditions [66]. However, both single-vessel models present a drawback, as they do not simulate peristaltic movements and exhibit significant static characteristics, which do not adequately represent dynamic changes in the human intestine [87,91]. As a result, single-vessel models offer a cost-effective solution for research; however, they have limited capacity to replicate GI tract conditions, thus restricting their applications.

To address these limitations, researchers have developed models based on multi-vessel bioreactors, such as SHIME, TIM (1 and 2), SIMGI and 3-ECSIM. The TIM and SIMGI models provide an advantage over SHIME by using peristaltic motion to mix the luminal content [71,84]. As indicated in [84], the operational parameters of SIMGI are based on the original SHIME description [73]. Therefore, not only the number of vessels but also other operational parameters, such as pH, are consistent between the two setups [84,122,123]. On the other hand, TIM-2 models present a considerable advantage over these models in data collection efficiency because the TIM models have a total working time of approximately one week, whereas the SHIME models necessitate an experimental duration of several weeks [71,73,78]. Also, each TIM-2 unit can be inoculated with distinct faecal samples, facilitating the comparative analysis of diverse microbiomes [71]. However, the modified SHIME model offers a significant advantage over TIM systems as it allows the investigation of both luminal and mucosal bacteria through two separate units operating under identical conditions. The combined M-SHIME and TWIN-SHIME models facilitate the study of specific bacteria, such as Lactobacilli species, which are exclusively found in the mucosal environment [124]. The SIMGI model offers a significant advantage over TIM models by enabling the linkage of different GI parts. This feature allows for the simultaneous study of food digestion in the stomach and small intestine and the investigation of the fermentation process in the proximal colon [84]. However, the disadvantage of the SIMGI model is that it requires continuous flushing with nitrogen [71]. Consequently, the derivative of P-ECSIM model 3S-ECSIM boasts the distinct advantage of creating anoxic conditions through microbiota metabolism [87].

Various high-throughput models have been developed to address the limitations of high costs and long culture times. This review examined the MimiCol (and its derivative, MimiCol^3^), MiPro model and gut on a chip as potential solutions. The primary distinction between these high-throughput models lies in the scale, as the MimiCol^3^ model features three vessels, each with a capacity of 150 mL to 250 mL, MiPro model uses a 96-well plate, with a typical volume of 1 mL per well; meanwhile, microfluidic chips typically range from a few square millimetres to square centimetres and work on the microlitre scale [68,92,94,125]. The MimiCol model is specifically designed to replicate the functions of the ascending colon. In contrast, the gut-on-a-chip model is utilised in various iterations such as the intestine-on-a-chip, colon-on-a-chip and gut-microbiome-on-a-chip models [91,101,126,127]. The integration of microfluidics technology provides the benefit of replicating various in vivo characteristics, such as peristaltic movements, intestinal barrier, mass transport and shear forces [127]. The microfluidics-based HMI platform can also be connected to the SHIME system. The HMI module simulates not only the dynamic conditions of the gut but also includes enterocytic cell lines to better understand host–microbiome interactions [88].

Despite the significant benefits discussed in this review, a substantial research gap persists in microbiome inclusion in in vitro models of the diseases. The human microbiome’s complexity, involving several thousands of different bacterial taxonomic units and millions of links between them [128], presents a formidable challenge in developing accurate and comprehensive models. Furthermore, the variability in microbiome composition among individuals, influenced by genetics, diet, environment, lifestyle or even personality traits, complicates creating a universally applicable in vitro model [129,130]. The dynamic and bidirectional interactions between microbes and their human hosts add complexity, as they profoundly impact host physiology, immune responses and disease processes [131]. Overcoming these challenges demands advanced methodologies to fully exploit the potential of microbiome-based models in biomedical research. Integrating human cell- and microbiome-based models presents a significant opportunity for gaining a more comprehensive understanding of GI pathophysiology. These systems facilitate the exploration of diverse aspects of diseases and the evaluation of treatment effects, thereby enhancing our comprehension. Incorporating the human gut microbiome into bioreactor-based models involving not only eukaryotic cells but also human gut microbiome can provide a deeper understanding of the impact of bacteria and their metabolites on diseases, thereby filling a crucial gap in our understanding of disease initiation and progression. It is important to underscore that the wider adoption of bioreactor-based models has the potential to decrease the necessity for animal studies, thereby endorsing more ethical and accurate research methodologies. The rapid development of this field has the potential to advance personalised medicine by creating “digital twins” of cancer cells and microbiomes. These models could be utilised to simulate the progression of the disease and predict a patient’s response to treatment with a high degree of specificity, enabling better treatment decision-making in clinical practice [132]. However, it is important to note the limitations of this review paper. While our study primarily utilised the PubMed database for its comprehensive coverage and sophisticated search methods, future research could benefit from including additional databases such as Web of Science and the Cochrane Database to enhance the breadth and validation of the findings.

## 5. Conclusions

This review highlights significant advancements in the in vitro modelling of gastrointestinal cancer, mainly through bioreactor-based systems. Our analysis underscores the potential of these models to elucidate the complex interactions between cancer treatments and the stool microbiome, including investigation of side effects such as radiation-induced toxicity, chemotherapy-induced dysbiosis and medication or surgical impacts on the gut microbiome oralisation processes. These bioreactor-based systems, which integrate stool microbiomes, represent a crucial step towards enhancing personalised medicine in oncology by providing more tailored and accurate insights into treatment effects. Because it is an emerging field in oncology, future studies should focus on refining these models and exploring additional variables to realise their potential in clinical applications fully.

## Data Availability

Not applicable.

## Supplementary Materials

The following supporting information can be downloaded at: https://www.mdpi.com/article/10.3390/cancers16173113/s1, Table S1: summarised characteristics of the analysed in vitro systems [133,134,135,136,137,138,139,140,141,142,143,144,145,146,147,148,149,150,151].
