# Supplementary material for: Cellular and Microbial In Vitro Modelling of Gastrointestinal Cancer
Source: Cancers (Basel). 2024 Sep 9;16(17):3113. doi: 10.3390/cancers16173113 (PMC11394127; doi:10.3390/cancers16173113)
Supplement: Supplementary file 1 [file cancers-16-03113-s001.zip › cancers-3128599-supplementary - updated.pdf]

Table S1. Summarized characteristics of the analyzed in vitro systems.

	Simulated GI Part	Operation Mode	Working Volume	No. of Vessels	How many experiments can be run at the same time?	Agitation speed	Mixing	pH	Temperature (⁰C)	Residence /Retention* Time	Microbial stabilization time	Total Working Time	Mucus Layer	Cell Lines	Peristaltic Pumping	Gas Mixture	Gassing Mode	Inoculum	Advantages	Disadvantages	Reference
Single-vessel models																					
**Batch Fermenter**	Any GI tract region [1,2]	Batch [1,2]	70-280 mL, but can vary [1,2]	1 [1]	1 [1,2]	300 rpm [1]	Mixed constantly with magnetic stirrers [1,2]	6.5-7.0 [1,2]	37 [1,2]	24 h [1,2]	Not specified	≤48 h [1,2]	No [1,2]	No [1,2]	No [1,2]	N_2 and CO_2 (80:20) or oxygen-free N_2 [1,2]	Flushing [1]	Faecal sample / 5% (w/v) Faecal slurry [1,2]	1. Fast, easy to maintain, reproducible<br>2. Closed system - low risk of contamination<br>3. Low cost [1,2]	1. Short culture periods, as nutrients decrease over time<br>3. Growth inhibition of the build-up of the waste products<br>4. Do not reflect stable *in vivo* conditions<br>6. Lack of environmental control [1,2]	[1,2]
**P-EC-SIM**	Proximal colon [3,4]	24-h batch culture, followed by continuous cultivation [4]	2 L [4]	1 [4]	1 [4]	400 rpm [4]	Stirring [4]	5.75 [4]	37 [4]	12.5 h (short retention time) to 25 h (long retention time) [4]		Several days [3,4]	No [4]	No [4]	No [4]	N_2, H_2 and CO_2 [4]	Initial flushing with N_2, anaerobic conditions maintained by bacterial metabolism [4]	20% (w/v) faecal slurry [4]	Self-maintained anaerobic conditions [4]	1. Only simulates proximal colon<br>2. Should be inoculated with luminal content from a human proximal colon; proximal bacteria and bacteria in faecal sample might not be the same. [4]	[3,4]

**MimiCol**	Ascending colon [5]	Semi-continuous[5]	150 mL [5]	1 [5]	1 [5]	6 rpm [5]	Continuous mixing through the integrated stirring system [5]	6.2 ± 0.25, starting at 7.4 [5]	37 [5]		1 h [5]	8 h [5]	No [5]	No [5]	No [5]	N_2 [5]	Headspace gassing [5]	Stool [5]	1. Smal volume<br>2. Closer simulation of in vivo mixing patterns [5]	Only 1 vessel [5]	[40]
Multi-vessel models																					
**SHIME**	Duodeum and jejunum (V1);<br>Ileum (V2);<br>Caecum and Ascending colon (V3);<br>Transverse colon (V4);<br>Descending colon (V5) [6]<br><br>Stomach (V1);<br>Small intestine (V2);<br>Ascending colon (V3);<br>Transverse colon (V4);<br>Descending colon (V5) [7]	Semi-continuous [6]	V1 = 300 mL<br>V2 = 300 mL<br>V3 = 1000 mL<br>V4 = 1600 mL<br>V5 = 1200 mL [6]<br><br>V1 = 200 mL<br>V2 = 300 mL<br>V3 = 700 mL<br>V4 = 1300 mL<br>V5 = 800 mL [7]<br><br>V2 = 200 mL<br>V3 = 500 mL<br>V4 = 800 mL<br>V5 = 600 mL [8]	5 [6]	Simulation of one colon region of 5 different donors or 5 different colon regions can be studied with one microbiome [6,7]	150 rpm [6]	Mixed constantly with magnetic stirrers [6]	V1 = 2.0-2.5<br>V2 = 5.0-6.0<br>V3 = 5.5-6.0<br>V4 = 6.0-6.4<br>V5 = 6.6-6.9 [7]<br><br>V3 = 5.6-5.9<br>V4 = 6.1-6.4<br>V5 = 6.6-6.9 [8]	37 [6]	V1 = 2 h<br>V2 = 6 h<br>V3 = 18 h<br>V4 = 36 h<br>V5 = 22 h [7]<br><br>V1 = 4 h<br>V2 = 4 h<br>V3 = 20 h<br>V4 = 32 h<br>V5 = 24 h [6]	~3 weeks [7,9]	~6 weeks [6]	No [6–9]	No [6–9]	Yes, the first two vessels [8]	N_2 [6,7]	No gas exchange between the different vessels;<br>The headspace of the culture system is flushed twice a day for 15 min. with gas [7]	20% Faecal suspension derived from one donor;<br>Pooled sample from 5 donors [6,7]	1. Simulates entire GI tract<br>2. Allows colon-region-specific research<br>3. Maintains microbiome stability over a long period<br>4. Differentiation between mucosal and luminal microbiome (M-SHIME)<br>5. Parallel control and treatment in TWIN-SHIME setup<br>6. Can be combined with HMI module [9,10]	1. Lack of dialysis;<br>2. Lack of peristalsis;<br>3. Lack of epithelial or immune cells [9]<br>4. Lack of absorption of metabolites [6]	[6–10]
**TWIN-SHIME**	Stomach (V1);<br>Small intestine (V2);<br>Ascending	Semi-continuous [11]	V3 = 500 mL<br>V4 = 800 mL	Two units with 5 vessels	Two parallel experiments [9]		Continuously stirred [12]	V1 = 2<br>V2 = 6.6<br>V3 = 5.6<br>V4 = 6.15<br>V5 = 6.6 [13]	37°C [11]	Total retention time of 72 h [12]	10-20 days after inoculation [13]	6 weeks [13]	Luminal phase only [13]	No	No	N_2 [12]	Flushing [12]	Stool [12]	1. The number of vessels (and therefore the colon parts) can be varied;	No mucosal layer (Liu et al., 2018	[9,11–13]

	colon (V3); Transverse colon (V4); Descending colon (V5) [11,12]		V5 = 600 mL [13]	each [11]															2. 2 parallel systems allow identical environmental conditions and identical microbial composition; 3. Combination with the M-Shime model is possible [11]		
**M-SHIME**	Stomach (V1); Small intestine (V2); Ascending colon (V3); Transverse colon (V4); Descending colon (V5) [11,14]	Semi-continuous [11]	V1 = 200 mL V2 = 300 mL V3 = 700 mL V4 = 1300 mL V5 = 800 mL [7,15]	3-5 [11,14]	2-3 parallel systems possible [13,14]	200 rpm [16]		5.6–5.9 [16]	37 °C [17]		10-20 days after inoculation [13]	Several weeks (8-12 weeks)	Yes [13]	No	No	N_2 [12,17]	Flushing with N_2 twice per day for 5-15 min [16,17]	Stool [11,14]	Simulation of the mucosal microbiome [11]	No peristaltic pumping	[11,13–19]
**TIM-1**	Stomach (V1); Duodenum (V2); Jejunum (V3); Ileum (V4) [20,21]	Continuous followed by fed batch [21]	V1 = 300 mL V2 = 55 mL V3 = 130 mL V4 = 130 mL [9]	4 [20,21]	1 [20]		Flexible membrane contracts and causes peristaltic waves [9,21]	pH programmed as gradient over time from 2.0 in the stomach, 5.8 in proximal colon, 6.4 in transverse colon, to 7.0 in distal colon	37 [20,22]		12-16 h [9]	Approximately one week	No	No	Yes [21]	N_2 [21]		Stool [15]	Peristaltic movements [20,21]	No simulation of cell interactions [20,21]	[9,15,20–24]
**TIM-2**	Proximal colon or entire colon [9]	Continuous followed by fed batch [25]	200 mL [25]	4 [9]	1 Hatanaka et al., 2012]		Flexible membrane contracts and causes peristaltic waves [9]	pH programmed as gradient over time from 2.0 in the stomach, 5.8 in proximal colon, 6.4 in transverse colon, to 7.0 in distal colon	37 [25]		12-16 h [9,26]	Approximately one week [9,26]	No [25]	No [25]	Yes [25]	N_2 [25]	Flushing with N_2 overnight [25]	Stool [9]	1. Peristaltic movements [25] 2. Dialysis; 3. Short experiment duration; 4. No phase-separation of solids and liquids [9]		[9,25–30]
**SIMGI**	Stomach Small intestine	Continuous or semi-	AC = 250 mL TC = 400	5 [9]	1 [9]	150 rpm [31]	Using a magnetic stirrer	Intestinal content: 6.8 AC: 5.6 ± 0.2	37 [9]	The overall residence	14 days [9]	45 days [32]	No	No	Yes [9]	N_2 [9]	Continuous flushing [9]	Stool [9]	1. Long period of cultures;	1. Complex operation to	[9,31,32]

	Large intestine (colon): Ascending Colon (AC), Transverse Colon (TC), Descending Colon (DC) [9]	continuous feeding 1-6 times/day [31]	mL DC = 300 mL [9]				and peristaltic movements [9,31]	TC: 6.3 ± 0.2 DC: 6.8 ± 0.2 [9,31]		time of 76 h [9,31]									2. Allows sampling at different system zones. 3. Gastric content is mixed by peristaltic movements 4. Controlled emptying of the gastric and small intestine compartments 5. Automated control of the working parameters 6. Compartments can be connected, or digestion and fermentation can run independently 7. Different operation modes due to software system possible [9]	keep anaerobiosis; 2. Lack of devices to evaluate the formation of microbial biofilms [9]	
**3S-EC-SIM**	Proximal (R1); Transversal (R2); Distal (R3) [3,33]	24 h batch, followed by continuous culture [33]	1 L [3]	3 [3,33]	1 (Brugère et al., 2011	40-1000 rpm (Brugère et al., 2011	Pendular motion using a Rushton turbine and a marine propeller (Brugère et al., 2011	R1 = 5.7 R2 = 6.2 R3 = 6.8 [33]	37 [4,33]	R1 = 12.5 h R2 = 17.7 h R3 = 17.7 h [33]	240 h [33]	Several days/weeks [33]	No [3,4,33]	No [3,4,33]	Bioreactors R1 was continuously fed using a multihead peristaltic pump; Multihead peristaltic pumps also used to transfer	N_2 [33]	Flushed with N_2 at the beginning, anoxic environment originating from the microbiota itself [33]	Stool [4,33]	1. Mimics different parts of the colon; 2. Conditions in each of the bioreactors can be modified; 3. Can simulate bacterial biofilms; 4. An anaerobic atmosphere is maintained by the microbiota metabolism [3,33]	No mucosal layer [33]	[3,4,33]

															medium from R1 to R2 and from R2 to R3 [33]						
**HMI**	HMI: two set ups with each two compartments: a) Upper compartment: luminal side of GIT b) Lower compartment contains enterocytes [10]		Varies, as can be connected to SHIME	Two-compartment reactor connected to SHIME (adapted version) [10,34,35]	2 [10]		Continuously stirred [10]	HMI module: 6.8 Ascending Colon: 5.6–5.9 [8,10]	37 [10]	20 h [8]		48 h [10]	Yes [10]	Enterocytes [10]	Yes [10]	Microaerophilic conditions, 95% N_2 & 5% CO_2 (Marzorati et al., 2014	a) Anaerobic upper chamber (water previously gassed with 95% N_2 & 5% CO_2) b) Aerobic lower chamber (liquid constantly gassed with air pump) [10]	Stool [10]	1. Possibility to simulate bacterial adhesion to the gut; 2. Possibility to couple it with existing bioreactor systems, e.g., SHIME; 3. Co-culturing of gut-representative microbial community with enterocyte-like cells; 4. Possibility to study the biofilms [10]		[10,34,35]
**EnteroMix**	Ascending (V1); transverse (V2); descending (V3); sigmoidal (V4) colon [36,37]		V1 = 3 mL; V2 = 5 mL; V3 = 7 mL; V4 = 9 mL [36,37]		4 parallel system sets [36,37]		No [36,37]	V1 = 5.5; V2 = 6.0; V3 = 6.5; V4 = 7.0 [36,37]	37 [36,37]	72 hours [36,37]	24 hours [36,37]	2 days [36,37]	No [36,37]	No [36,37]	No [36,37]	N_2 [36,37]	Flushing [36,37]	Stool [36,37]	1. Uses very low volume of the medium; 2. Ability to run four parallel experiments using the same faecal sample as inoculum. [36,37]	1. Lack of temperature control through the water jacket; 2. No simulation of digestion and absorption of nutrients; 3. No host cells. 4. Only short-term experiments possible [36,37]	[36,37]
**PolyFermS**	Proximal colon [38]		140-300 mL [38,39]	Multiple [38]			Yes [38]	5.5 [38]	37 [38]			6 days [38]	No[38]	No [38]	Yes [38]	CO_2 [38]		Stool [38]	1. High reproducibility and biological replication;	Higher risk of contamination [38]	[38]

																			2. Possibility to test different treatments on the same microbiota [38]		
High-throughput models																					
**MimiCol^{3}**	Ascending colon [40]	Semi-continuous [40]	150 mL [40] Each vessel has the capacity of 250 mL [41]	3 [40]	3 experiments [40]	100 rpm [40]	Continuous mixing through the integrated stirring system [41]	6.2 ± 0.25, starting at 7.4 [40,41]	37 [40,41]			9-24 h [40,41]	No [40,41]	No [40,41]	Yes [41]	N_2 [40]	Headspace gassing [40]	Stool [40,41]	1. Simple experimental conditions; 2. Short experimental time; 3. Three vessels are used in parallel, but control of the vessels is independent; 4. Vessel and lid parts can be exchanged or sterilized easily [40,41]	Only one part of the GI tract is simulated [40]	[40,41]
**MiPro**	Any part of GI tract	Semi-continuous	1 mL	96 deep-well plate	Depends on the experimental setup	500 rpm	Continuous mixed using a shaker	Not specifies	37			48 hours	No	No	No	5% H_2, 5%CO_2, and 90% N_2	In anaerobic workstation	2% (w/v) faecal slurry	High throughput, no specific instruments is needed, simple approach	Limited possibilities to monitor and control process parameters	[42]
**Gut-on-a-Chip**	Colon, intestine [43,44]		700 μL					Not controlled				5 h-14 d. [45]	Yes [45]	Yes [45]	Yes [45]			Stool Caco-2 Stem cells [45] Combination of different cell types: immune cells, intestinal epithelial cells, vascular endothelial cells, *E. Coli*, *Lactobacillus*	1. Reproduces relevant characteristics of the gut: intestinal barrier, oxygen gradient, peristalsis, ability to create cyclic motion, shear stress, and mass transport [45,46] 2. 3D modulation [45] 3. Recapitulates gut hydrodynamic conditions [45] 4. Co-culturing of different cell lines [45]	1. A lifetime of tissue and microbial cultures is limited; 2. Specific fabric materials like artificial polymers are needed; 3. Difficult to maintain all cells together and provide the different required growth media and microenvironmental	[43–46]

																		rhamnosus GG and Bac- teroides caccae [46]		conditions [45]	

Reference

1. Takagi, R.; Sasaki, K.; Sasaki, D.; Fukuda, I.; Tanaka, K.; Yoshida, K.-I.; Kondo, A.; Osawa, R. A Single-Batch Fermentation System to Simulate Human Colonic Microbiota for High-Throughput Evaluation of Prebiotics. *PLoS ONE* **2016**, *11*, e0160533. https://doi.org/10.1371/journal.pone.0160533.
2. Wang, X.; Gibson, G.R. Effects of the in Vitro Fermentation of Oligofructose and Inulin by Bacteria Growing in the Human Large Intestine. *J. Appl. Bacteriol.* **1993**, *75*, 373–380. https://doi.org/10.1111/j.1365-2672.1993.tb02790.x.
3. Brugère, J.-F.; Féria-Gervasio, D.; Popse, Z.; Tottey, W.; Alric, M. The ECSIM Concept (Environmental Control System for Intestinal Microbiota) and Its Derivative Versions to Help Better Understand Human Gut Biology. *Appl. Biomed. Eng.* **2011**, *4*, 63–82.
4. Feria-Gervasio, D.; Denis, S.; Alric, M.; Brugère, J.-F. In Vitro Maintenance of a Human Proximal Colon Microbiota Using the Continuous Fermentation System P-ECSIM. *Appl. Microbiol. Biotechnol.* **2011**, *91*, 1425–1433. https://doi.org/10.1007/s00253-011-3462-5.
5. Beeck, R.; Glöckl, G.; Krause, J.; Schick, P.; Weitschies, W. Mimicking the Dynamic Colonic Microbiota in Vitro to Gain a Better Understanding on the in Vivo Metabolism of Xenobiotics: Degradation of Sulfasalazine. *Int. J. Pharm.* **2021**, *603*, 120704. https://doi.org/10.1016/j.ijpharm.2021.120704.
6. Molly, K.; Vande Woestyne, M.; Verstraete, W. Development of a 5-Step Multi-Chamber Reactor as a Simulation of the Human Intestinal Microbial Ecosystem. *Appl. Microbiol. Biotechnol.* **1993**, *39*, 254–258. https://doi.org/10.1007/BF00228615.
7. De Boever, P.; Deplancke, B.; Verstraete, W. Fermentation by Gut Microbiota Cultured in a Simulator of the Human Intestinal Microbial Ecosystem Is Improved by Supplementing a Soygerm Powder. *J. Nutr.* **2000**, *130*, 2599–2606. https://doi.org/10.1093/jn/130.10.2599.
8. Possemiers, S.; Verthé, K.; Uyttendaele, S.; Verstraete, W. PCR-DGGE-Based Quantification of Stability of the Microbial Community in a Simulator of the Human Intestinal Microbial Ecosystem. *FEMS Microbiol. Ecol.* **2004**, *49*, 495–507. https://doi.org/10.1016/j.femsec.2004.05.002.
9. Verhoeckx, K.; Cotter, P.; López-Expósito, I.; Kleiveland, C.; Lea, T.; Mackie, A.; Requena, T.; Swiatecka, D.; Wichers, H. (Eds.) *The Impact of Food Bioactives on Health*; Springer International Publishing: Cham, Switzerland, 2015; ISBN 978-3-319-15791-7.
10. Marzorati, M.; Vanhoecke, B.; De Ryck, T.; Sadaghian Sadabad, M.; Pinheiro, I.; Possemiers, S.; Van den Abbeele, P.; Derycke, L.; Bracke, M.; Pieters, J.; et al. The HMI™ Module: A New Tool to Study the Host-Microbiota Interaction in the Human Gastrointestinal Tract in Vitro. *BMC Microbiol.* **2014**, *14*, 133. https://doi.org/10.1186/1471-2180-14-133.
11. Van den Abbeele, P.; Roos, S.; Eeckhaut, V.; MacKenzie, D.A.; Derde, M.; Verstraete, W.; Marzorati, M.; Possemiers, S.; Vanhoecke, B.; Van Immerseel, F.; et al. Incorporating a Mucosal Environment in a Dynamic Gut Model Results in a More Representative Colonization by Lactobacilli. *Microb. Biotechnol.* **2012**, *5*, 106–115. https://doi.org/10.1111/j.1751-7915.2011.00308.x.
12. Van den Abbeele, P.; Grootaert, C.; Marzorati, M.; Possemiers, S.; Verstraete, W.; Gérard, P.; Rabot, S.; Bruneau, A.; El Aidy, S.; Derrien, M.; et al. Microbial Community Development in a Dynamic Gut Model Is Reproducible, Colon Region Specific, and Selective for Bacteroidetes and Clostridium Cluster IX. *Appl. Env. Microbiol.* **2010**, *76*, 5237–5246. https://doi.org/10.1128/AEM.00759-10.
13. Liu, L.; Firrman, J.; Tanes, C.; Bittinger, K.; Thomas-Gahring, A.; Wu, G.D.; Van den Abbeele, P.; Tomasula, P.M. Establishing a Mucosal Gut Microbial Community in Vitro Using an Artificial Simulator. *PLoS ONE* **2018**, *13*, e0197692. https://doi.org/10.1371/journal.pone.0197692.
14. Liu, Y.; Matheyambath, A.C.; Polic, I.I.; LaPointe, G. Differential fermentation of raw and processed high-amylose and waxy maize starches in the Simulator of the Human Intestinal Microbial Ecosystem (SHIME®). *J. Funct. Foods* **2021**, *86*, 104735, https://doi.org/10.1016/j.jff.2021.104735.
15. Roussel, C.; De Paepe, K.; Galia, W.; De Bodt, J.; Chalancon, S.; Leriche, F.; Ballet, N.; Denis, S.; Alric, M.; Van de Wiele, T.; et al. Spatial and temporal modulation of enterotoxigenic E. coli H10407 pathogenesis and interplay with microbiota in human gut models. *BMC Biol.* **2020**, *18*, 1–21, https://doi.org/10.1186/s12915-020-00860-x.
16. Vanlancker, E.; Vanhoecke, B.; Stringer, A; Van de Wiele, T. 5-Fluorouracil and irinotecan (SN-38) have limited impact on colon microbial functionality and composition in vitro. *PeerJ*. 2017, *5*, e4017.doi: 10.7717/peerj.4017.

17. Van den Abbeele, P.; Belzer, C.; Goossens, M.; Kleerebezem, M.; De Vos, W.M.; Thas, O.; De Weirdt, R.; Kerckhof, F.-M.; Van de Wiele, T. Butyrate-Producing Clostridium Cluster XIVa Species Specifically Colonize Mucins in an in Vitro Gut Model. *ISME J.* **2013**, *7*, 949–961. https://doi.org/10.1038/ismej.2012.158.
18. Arroyo, M.C.; Laurie, I.; Rotsaert, C.; Marzorati, M.; Risso, D.; Karnik, K. Age-Dependent Prebiotic Effects of Soluble Corn Fiber in M-SHIME® Gut Microbial Ecosystems. *Plant Foods Hum. Nutr.* **2023**, *78*, 213–220, https://doi.org/10.1007/s11130-023-01043-z.
19. Nissen, L.; Casciano, F.; Gianotti, A. Intestinal fermentation *in vitro* models to study food-induced gut microbiota shift: an updated review. *FEMS Microbiol. Lett.* **2020**, *367*, https://doi.org/10.1093/femsle/fnaa097.
20. Hatanaka, M.; Nakamura, Y.; Maathuis, A.; Venema, K.; Murota, I.; Yamamoto, N. Influence of Bacillus subtilis C-3102 on microbiota in a dynamic in vitro model of the gastrointestinal tract simulating human conditions. *Benef. Microbes* **2012**, *3*, 229–236, https://doi.org/10.3920/bm2012.0016.
21. Minekus, M.; Marteau, P.; Havenaar, R.; Veld, J.H.J.H. in't A Multicompartmental Dynamic Computer-Controlled Model Simulating the Stomach and Small Intestine. *Altern. Lab. Anim.* **1995**, *23*, 197–209. https://doi.org/10.1177/026119299502300205.
22. Dickinson, P.A.; Abu Rmaileh, R.; Ashworth, L.; Barker, R.A.; Burke, W.M.; Patterson, C.M.; Stainforth, N.; Yasin, M. An Investigation into the Utility of a Multi-compartmental, Dynamic, System of the Upper Gastrointestinal Tract to Support Formulation Development and Establish Bioequivalence of Poorly Soluble Drugs. *AAPS J.* **2012**, *14*, 196–205, https://doi.org/10.1208/s12248-012-9333-x.
23. Minekus, M. The TNO Gastro-Intestinal Model (TIM). In *The Impact of Food Bioactives on Health*; Verhoeckx, K., Cotter, P., López-Expósito, I., Kleiveland, C., Lea, T., Mackie, A., Requena, T., Swiatecka, D., Wichers, H., Eds.; Springer International Publishing: Cham, Switzerland, 2015; pp. 37–46, ISBN 978-3-319-15791-7.
24. Venema, K.; Verhoeven, J.; Verbruggen, S.; Espinosa, L.; Courau, S. Probiotic survival during a multi-layered tablet development as tested in a dynamic, computer-controlled in vitro model of the stomach and small intestine (TIM-1). *Lett. Appl. Microbiol.* **2019**, *69*, 325–332.
25. Minekus, M.; Smeets-Peeters, M.; Bernalier, A.; Marol-Bonnin, S.; Havenaar, R.; Marteau, P.; Alric, M.; Fonty, G.; Huis in't Veld, J.H. A Computer-Controlled System to Simulate Conditions of the Large Intestine with Peristaltic Mixing, Water Absorption and Absorption of Fermentation Products. *Appl. Microbiol. Biotechnol.* **1999**, *53*, 108–114. https://doi.org/10.1007/s002530051622.
26. Maas, E.; Penders, J.; Venema, K. Studying Fungal-Bacterial Relationships in the Human Gut Using an In Vitro Model (TIM-2). *J. Fungi* **2023**, *9*, 174, https://doi.org/10.3390/jof9020174.
27. Bordonaro, M.; Venema, K.; Putri, A.K.; Lazarova, D. Approaches that ascertain the role of dietary compounds in colonic cancer cells. *World J. Gastrointest. Oncol.* **2014**, *15*, 1–10.
28. Gao, K.; Xu, A.; Krul, C.; Venema, K.; Liu, Y.; Niu, Y.; Lu, J.; Bensoussan, L.; Seeram, N.P.; Heber, D.; et al. Of the Major Phenolic Acids Formed during Human Microbial Fermentation of Tea, Citrus, and Soy Flavonoid Supplements, Only 3,4-Dihydroxyphenylacetic Acid Has Antiproliferative Activity. *J. Nutr.* **2006**, *136*, 52–57, https://doi.org/10.1093/jn/136.1.52.
29. Kong, H.; Wang, M.; Venema, K.; Maathuis, A.; van der Heijden, R.; van der Greef, J.; Xu, G.; Hankemeier, T. Bioconversion of red ginseng saponins in the gastro-intestinal tract in vitro model studied by high-performance liquid chromatography–high resolution Fourier transform ion cyclotron resonance mass spectrometry. *J. Chromatogr. A* **2009**, *1216*, 2195–2203, https://doi.org/10.1016/j.chroma.2008.11.030.
30. Rehman, A.; Heinsen, F.-A.; E Koenen, M.; Venema, K.; Knecht, H.; Hellmig, S.; Schreiber, S.; Ott, S.J. Effects of probiotics and antibiotics on the intestinal homeostasis in a computer controlled model of the large intestine. *BMC Microbiol.* **2012**, *12*, 47–47, https://doi.org/10.1186/1471-2180-12-47.
31. Barroso, E.; Cueva, C.; Peláez, C.; Martínez-Cuesta, M.C.; Requena, T. Development of Human Colonic Microbiota in the Computer-Controlled Dynamic SIMulator of the GastroIntestinal Tract SIMGI. *LWT Food Sci. Technol.* **2015**, *61*, 283–289. https://doi.org/10.1016/j.lwt.2014.12.014.
32. Gil-Sánchez, I.; Cueva, C.; Tamargo, A.; Quintela, J.C.; de la Fuente, E.; Walker, A.W.; Moreno-Arribas, M.V.; Bartolomé, B. Application of the dynamic gastrointestinal simulator (simgi®) to assess the impact of probiotic supplementation in the metabolism of grape polyphenols. *Food Res. Int.* **2019**, *129*, 108790, https://doi.org/10.1016/j.foodres.2019.108790.
33. Feria-Gervasio, D.; Tottey, W.; Gaci, N.; Alric, M.; Cardot, J.-M.; Peyret, P.; Martin, J.-F.; Pujos, E.; Sébédio, J.-L.; Brugère, J.-F. Three-Stage Continuous Culture System with a Self-Generated Anaerobia to Study the Regionalized Metabolism of the Human Gut Microbiota. *J. Microbiol. Methods* **2014**, *96*, 111–118. https://doi.org/10.1016/j.mimet.2013.11.015.
34. Venema, K.; van den Abbeele, P. Experimental Models of the Gut Microbiome. *Best. Pract. Res. Clin. Gastroenterol.* **2013**, *27*, 115–126. https://doi.org/10.1016/j.bpg.2013.03.002.
35. von Martels, J.Z.; Sadabad, M.S.; Bourgonje, A.R.; Blokzijl, T.; Dijkstra, G.; Faber, K.N.; Harmsen, H.J. The role of gut microbiota in health and disease: In vitro modeling of host-microbe interactions at the aerobe-anaerobe interphase of the human gut. *Anaerobe* **2017**, *44*, 3–12, https://doi.org/10.1016/j.anaerobe.2017.01.001.
36. Makivuokko, H.; Nurmi, J.; Nurminen, P.; Stowell, J.; Rautonen, N. In Vitro Effects on Polydextrose by Colonic Bacteria and Caco-2 Cell Cyclooxygenase Gene Expression. *Nutr. Cancer* **2005**, *52*, 94–104, https://doi.org/10.1207/s15327914nc5201_12.

37. Williams, C.; Walton, G.; Jiang, L.; Plummer, S.; Garaiova, I.; Gibson, G. Comparative Analysis of Intestinal Tract Models. *Annu. Rev. Food Sci. Technol.* **2015**, *6*, 329–350, https://doi.org/10.1146/annurev-food-022814-015429.
38. Zihler Berner, A.; Fuentes, S.; Dostal, A.; Payne, A.N.; Vazquez Gutierrez, P.; Chassard, C.; Grattepanche, F.; de Vos, W.M.; Lacroix, C. Novel Polyfermentor Intestinal Model (PolyFermS) for Controlled Ecological Studies: Validation and Effect of PH. *PLoS ONE* **2013**, *8*, e77772. https://doi.org/10.1371/journal.pone.0077772.
39. Gościniak, A.; Eder, P.; Walkowiak, J.; Cielecka-Piontek, J. Artificial Gastrointestinal Models for Nutraceuticals Research—Achievements and Challenges: A Practical Review. *Nutrients* **2022**, *14*, 2560. https://doi.org/10.3390/nu14132560.
40. Beeck, R.; Dols, A.; Schneider, F.; Seradj, D.-S.; Krause, J.; Schick, P.; Weitschies, W. An Advanced Bioreactor Simulating Dynamic Physiological Conditions in the Human Ascending Colon: MimiCol3. *Pharmaceutics* **2022**, *14*, 1049. https://doi.org/10.3390/pharmaceutics14051049.
41. Seradj, D.-S.; Beeck, R.; Haase, A.; Krause, J.; Schick, P.; Weitschies, W. Influence of Different Diets on the Degradation of Sulfasalazine by Colon Bacteria Determined Using MimiCol3. *Pharmaceuticals* **2023**, *16*, 1128. https://doi.org/10.3390/ph16081128.
42. Li, L.; Abou-Samra, E.; Ning, Z.; Zhang, X.; Mayne, J.; Wang, J.; Cheng, K.; Walker, K.; Stintzi, A.; Figeys, D. An in Vitro Model Maintaining Taxon-Specific Functional Activities of the Gut Microbiome. *Nat. Commun.* **2019**, *10*, 4146. https://doi.org/10.1038/s41467-019-12087-8.
43. Bein, A.; Shin, W.; Jalili-Firoozinezhad, S.; Park, M.H.; Sontheimer-Phelps, A.; Tovaglieri, A.; Chalkiadaki, A.; Kim, H.J.; Ingber, D.E. Microfluidic Organ-on-a-Chip Models of Human Intestine. *Cell. Mol. Gastroenterol. Hepatol.* **2018**, *5*, 659–668, https://doi.org/10.1016/j.jcmgh.2017.12.010.
44. Sontheimer-Phelps, A.; Chou, D.B.; Tovaglieri, A.; Ferrante, T.C.; Duckworth, T.; Fadel, C.; Frismantas, V.; Sutherland, A.D.; Jalili-Firoozinezhad, S.; Kasendra, M.; et al. Human Colon-on-a-Chip Enables Continuous In Vitro Analysis of Colon Mucus Layer Accumulation and Physiology. *Cell. Mol. Gastroenterol. Hepatol.* **2020**, *9*, 507–526, https://doi.org/10.1016/j.jcmgh.2019.11.008.
45. Valiei, A.; Aminian-Dehkordi, J.; Mofrad, M.R.K. Gut-on-a-Chip Models for Dissecting the Gut Microbiology and Physiology. *APL Bioeng.* **2023**, *7*, 011502. https://doi.org/10.1063/5.0126541.
46. Marrero, D.; Pujol-Vila, F.; Vera, D.; Gabriel, G.; Illa, X.; Elizalde-Torrent, A.; Alvarez, M.; Villa, R. Gut-on-a-Chip: Mimicking and Monitoring the Human Intestine. *Biosens. Bioelectron.* **2021**, *181*, 113156. https://doi.org/10.1016/j.bios.2021.113156.
